# The Perception of Waiting Times on Patient Satisfaction and Patient Care: A Cross-Sectional Study at a Tertiary Health Care Institution in Kenya

**DOI:** 10.1371/journal.pone.0322015

**Published:** 2025-05-02

**Authors:** Soud Seif, Jasmit Shah, Ali Chandani, Sayed K. Ali

**Affiliations:** 1 Department of Medicine, Aga Khan University, Nairobi, Kenya; 2 Brain and Mind Institute, Aga Khan University, Nairobi, Kenya; CHUV: Centre Hospitalier Universitaire Vaudois, SWITZERLAND

## Abstract

**Introduction:**

Patients often spend significant time waiting for care, which influences patient satisfaction, perceived quality, access to care, and utilization of various health care services. Waiting time and resulting implications remain understudied in low- and middle-income countries like Kenya. This study aimed to describe the impact on the perception of waiting times on patient satisfaction and care at a tertiary healthcare institution in Kenya.

**Method:**

We conducted a prospective cross-sectional study between the first of April 2023 and the thirty-first of August 2023 at the Emergency Room (ER) at Aga Khan University Hospital, Nairobi. Data was collected through a structured questionnaire examining demographics and patient wait times. Descriptive statistics were summarized using frequencies and percentages, and univariate analyses using Fisher’s exact test were conducted for group comparisons.

**Results:**

A total of 941 patients participated in the study, with 52.0% being females and 53.6% in the 20–40-year age group. More than half of the patients were married (52.4%), had a university education (75.9%), and were employed (70.6%). Of the patients who took the survey, 51.1% reported waiting for 31–60 minutes, while 25.4% reported waiting for more than 60 minutes. Most patients presented to the ER on Monday, most frequently between 0800 and 1200 hrs. Most patients (70.8%) were likely to return for care and 71.7% were likely to recommend care at the ER to relatives and friends.

**Conclusion:**

Specific days and times of visits to the ER were associated with prolonged waiting times. To reduce waiting times and improve access to healthcare services, facilities should consider increasing the number of healthcare providers during these peak hours to ensure timely and quality consultations. Identifying bottlenecks and gridlocks within healthcare facilities is crucial to developing an efficient blueprint that aims to improve waiting times, leading to improved patient satisfaction and care.

## Introduction

Long wait times before patients see a doctor have become endemic in most healthcare settings. Patients generally spend a significant amount of time in hospitals waiting to receive care from healthcare providers. The extent to which patients are satisfied is widely thought to correlate with hospital waiting times [1]. Information regarding waiting times, the determining factors, and the impact of prolonged waiting times on the overall healthcare system, particularly in sub-Saharan Africa, including Kenya, remains sparse.

Healthcare organisations are constantly striving to improve patient satisfaction and experience. Patient satisfaction is a commonly used indicator for measuring the quality of healthcare services that most hospitals provide [2]. It also provides valuable information on how well providers meet patients’ expectations [3]. The perceived quality of healthcare is enhanced when services are delivered in a timely and efficient manner, with a focus on patient-centeredness [4]. Patient satisfaction is usually influenced by patient-provider interactions, including time spent with the physician, the physician’s willingness to listen to the patient, patient-specific characteristics such as expectations of care, and perceived waiting times [5]. Patient satisfaction, in turn, has been linked to positive outcomes such as improved compliance, reduced litigation, and better prognosis[6].

Waiting time is the time between a patient’s arrival at the hospital and when a healthcare practitioner sees them. This includes the time spent in the registration area, waiting room, and examination room before the arrival of the healthcare provider. Waiting time is an essential quality metric in patient experience surveys and a significant predictor of patient satisfaction [7]. Prolonged waiting times in hospitals have been negatively correlated with patient satisfaction and, ultimately, patient care [8]. Long waiting times have been the focus of increasing public attention and scrutiny, particularly in outpatient facilities. Prolonged waiting times lead to reduced access to care, disruption of workflows, and lower overall patient satisfaction [9]. While measuring quality metrics such as waiting times in low and middle-income countries (LMICs) may be challenging, studies have shown that well-designed surveys and questionnaires can provide reliable and objective data to measure quality and improve overall hospital performance [10,11].

The Institute of Medicine (IOM) recommends that healthcare providers see at least 90% of their patients within 30 minutes of their visit [12]. However, achieving this has been challenging, particularly in LMIC where resources are limited and healthcare systems remain fragile. Waiting times are a good indicator of quality of care and patient satisfaction. A study conducted in the Southern region of Ethiopia revealed that waiting times were longer than the recommended average, with more than half of the participants reporting dissatisfaction [7]. Similarly, a study in Nigeria showed that waiting times varied between 11 and 354 minutes, with the average waiting time being 146 minutes, influencing patient satisfaction and care [13].

Kenya is a country in East Africa with a population of around 55 million [14]. The Aga Khan University Hospital, Nairobi (AKUHN) is a 300-bed tertiary care facility in the capital city of Nairobi that offers advanced medical care to Kenyans and patients from neighbouring countries. Most patients admitted to the hospital are received through the 24-hour emergency room which is staffed by both consultant physicians and junior doctors in training.

This study was conducted to better understand the waiting time at the ER, its impact on patients’ experience, and whether there was a correlation between waiting time and patient’s satisfaction with the care they received.

## Materials and methods

### Study design

This was a prospective cross-sectional study conducted over 5 months between the first of April 2023 and the thirty-first of August 2023.

### Participants and study site

All patients over the age of 18 years seeking care at the ER at the Aga Khan University Hospital, Nairobi (AKUHN) were eligible to participate in the study. Current AKUHN ER policy dictates that all patients presenting at the ER are triaged based on the Canadian Triage and Acuity Scale (CTAS), a 5-point scale assessing the potential severity of a patient’s illness or injury with corresponding recommendations for time to physician contact. Patients scoring levels 1–3 on the CTAS (resuscitation, emergent and urgent) are assigned a higher level of acuity, are assigned to cubicles as opposed to being directed to the waiting area, and their time to be seen by a provider is independently tracked by the hospital as an ER outcome measure. Patients scoring levels 4–5 on the CTAS (less urgent and non-urgent)are not prioritized in a similar manner and their recommended waiting times are less than 60 minutes and less than 120 minutes respectively [15,16]. During the study period, 81% of the patients seen in the ER scored level 4–5 on the CTAS.

Based on the logistical difficulty of administering a survey to an emergent or urgent patient or one requiring active resuscitation when other processes take priority (collecting background and pertinent medical information, family members obtaining financial clearance, and patient and/or family stress levels) the authors decided to exclude patients scoring levels 1–3 on the CTAS for this study.

### Data collection

Data was gathered by a team of three research assistants, who received a one-hour face to face training by the primary research team in the administration of the survey and collection of data. The research assistants were primarily stationed within the ER during various times of the day and night. A convenience sampling strategy was used and patients were approached in the ER waiting area after triage but prior to receiving services. All participants were asked for written consent before taking a self-administered survey in either English or Kiswahili based on their preference. Family members or caregivers present with the participants were allowed to assist with the filling of the survey for patients with low level of literacy or disability. The trained research assistants were available to explain or clarify any ambiguity to patients, family members or caregivers accompanying the patients. Participants were followed through their ER visit and all surveys were completed prior to patient discharge from the ER or prior to hospital admission.

The survey used in this study was developed based on a literature review and discussion with the research team [17,18]. The questions included patients’ demographic information, perceived waiting times and overall satisfaction with the care provided. The collected data was entered into the Research Electronic Data Capture (REDCap) platform to facilitate analysis [19]. The English version of the survey was translated into Kiswahili by a group of linguistic experts and verified by the local research team.

### Statistical analysis

Categorical data were presented as frequencies and percentages. Fischer’s exact test was used to analyse the relationship between patient satisfaction and factors such as waiting time and time spent with the physicians. Furthermore, we used multivariate logistic regression to identify independent associations of satisfaction with waiting times and was presented with odds ratio (OR) and 95% confidence interval. The analysis was conducted using SPSS statistical software V.20.0 (IBM). The significance level was set at α=0.05, and all tests were two-tailed.

Approval for this study was obtained from the Institutional Scientific and Ethics Review Committee (ISERC) at the Aga Khan University, Nairobi [Ref: 2022/ISERC-103(v2)) and National Commission for Science Technology and Innovation (NACOSTI) (NACOSTI/P/23/23663).

### Ethics approval and consent

Approval for this study was obtained from the Institutional Scientific and Ethics Review Committee (ISERC) at the Aga Khan University, Nairobi and National Commission for Science Technology and Innovation (NACOSTI). All participants were informed about the study, and informed consent was obtained from all participants before conducting the study.

## Results

### Demographic characteristics

A total of 1300 surveys were handed out and 72% (941) completed surveys were collected. Of the 941 participants 52.0% (n = 489) were females. 53.6% (n = 504) of the participants were between 20 and 40 years old, and 29.3% (276) were in the 40–65 age group. Of the participants who completed the survey, 52.4% (n = 493) were married, 75.9% (n = 714) had a university education, and 70.6% (n = 664) were self-employed or employed. Most of the participants, 91.1%, were of African origin, with 77.0% (n = 725) living in Nairobi County and 14.8% (n = 139) from neighbouring Kiambu County. Table 1 summarises the overall socio-demographic characteristics of the participants.

**Table 1:** Socio-demographic characteristics of the participants.

Socio-demographic variables	N = 941
**Gender, n (%)**	
Male	452 (48.0)
Female	489 (52.0)
**Age in years, n (%)**	
< 20 years	83 (8.8)
20–40 years	504 (53.6)
40–65 years	276 (29.3)
> 65 years	78 (8.3)
**Marital status, n (%)**	
Single	372 (39.5)
Married	493 (52.4)
Others	76 (8.1)
**Level of education, n (%)**	
None	13 (1.4)
Primary	18 (1.9)
Secondary	161 (17.1)
Diploma	35 (3.7)
University	714 (75.9)
**Employment status, n (%)**	
Employed/ Self Employed	664 (70.6)
Not Employed	182 (19.3)
Retired	78 (8.3)
Student	17 (1.8)
**Race, n (%)**	
African	857 (91.1)
Others	84 (8.9)
**County, n (%)**	
Nairobi	725 (77.0)
Kiambu	139 (14.8)
Others	77 (8.2)

### Day and time of visits

**Fig 1:**
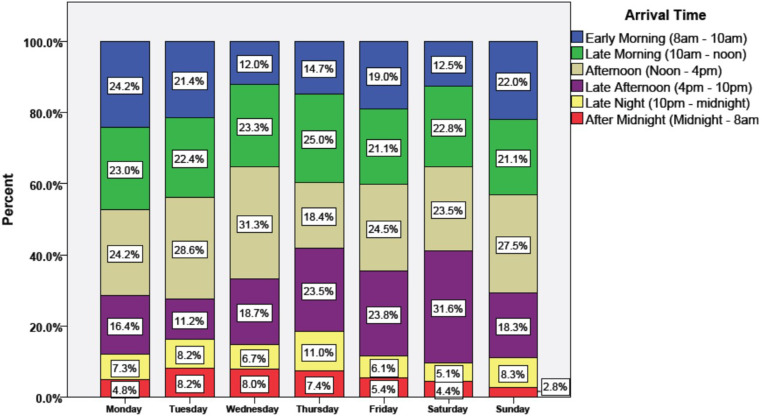
Participants day and time of visit.

The majority of the participants sought care in the ER on Monday (17.6%, n = 166), followed by Wednesday (15.9%, n = 150) and Friday (15.6%, n = 147). Tuesday had the least number of patients, accounting for only 10.4% (n = 98). Almost half of the patients (49.4%, n = 452) came to the ER between 10 am and 4 pm. The highest number of patients (47.2%) visited the ER in the early and late morning (8 am – 12 noon) on Mondays. Similarly, of those who visited the ER in the afternoon (Noon- 4 p.m.), Wednesday had the highest number of patients (31.3%). For a more detailed summary of the days and times of visit, please refer to Fig 1.

### Visit experience

76.5% (n = 720) of the study participants were new patients while 23.5% (n = 221) were patients who had previously visited the ER. 51.1% (n = 481) of the participants reported waiting 31–60 minutes to see a doctor, and 25.4% (n = 239) waited more than 60 minutes. Most participants (75.3%, n = 709) were happy or somewhat satisfied with the wait time. 72.9% (n = 686) of participants reported spending less than 15 minutes in their initial consultation with the doctor. Many participants (89.1%, n = 838) were happy or somewhat satisfied with the consultation time. The majority (91.7%, n = 863) of the participants reported that they would always or occasionally return to the hospital for care, and 91.9% (n = 865) stated that they would always or occasionally recommend the hospital to their relatives and friends. Table 2 summarises the overall experiences of the participants.

**Table 2:** Visit and Satisfaction characteristics of the participants.

	N = 941
**Purpose of Visit, n (%)**	
New Visit	720 (76.5)
Follow up	221 (23.5)
**On average how many times do you visit AKUH per year?, n (%)**	
None	151(16.0)
1 – 2	434(46.1)
3 – 5	277(29.4)
More than 5	79 (8.4)
**How much time did you wait to see a doctor (from registration to the time you saw the doctor), n (%)**	
Less than 30 minutes	221(23.5)
31-60 minutes	481(51.1)
61–120 minutes	233 (24.8)
>120 minutes	6 (0.6)
**Prolonged waiting time, n (%)**	239(25.4)
**How much time did you spend with the doctor in the consultation rooms initially, n (%)**	
Less than 15 minutes	686 (72.9)
More than 15 minutes	255 (27.1)
**Overall satisfaction score with wait times**	
**(From registration to when you saw the doctor), n (%)**	
Satisfied/ Somewhat Satisfied	709 (75.3)
Neutral	97 (10.3)
Not Satisfied/ Somewhat not Satisfied	135 (14.3)
**Overall satisfaction score with service times**	
**(Time spent in the consultation room with the doctor), n (%)**	
Satisfied/ Somewhat Satisfied	838 (89.1%)
Neutral	96 (10.2)
Not Satisfied/ Somewhat not Satisfied	7 (0.7)
**Likelihood to return for care at AKUH**	
Always	666 (70.8)
Occasionally	197 (20.9)
Rarely	63 (6.7)
Never	15 (1.6)
**Likelihood to recommend to relatives and friends**	
Always	675 (71.7)
Occasionally	190 (20.2)
Rarely	67 (7.1)
Never	9 (1.0)

### Association with prolonged waiting time

Based on the CTAS criteria for level 4 patients, the prolonged waiting time was defined as waiting for more that 60 minutes from registration to time seen by a healthcare provider [15, 16]. Of the participants who experienced long waiting times (> 60 minutes), 46.1% were over 40 years of age, 61.9% were married, and 74.1% were self-employed or employed. Mondays and Wednesdays had the longest waiting times; afternoons (12–4 pm) and evenings (4 pm-10 pm) were similarly associated with longer waiting times.

There was a statistically significant association between waiting times and overall satisfaction. Patients who experienced longer waiting times were more likely to be dissatisfied with their experience. On the other hand, patients who reported shorter waiting times (< 60 minutes) also reported a higher likelihood of returning for care and recommending the hospital to relatives and friends. Table 3 summarises the overall associations between various factors and prolonged waiting times.

**Table 3:** Associations between prolonged waiting times.

	Prolonged Waiting Time		P value
	< 60 mins	>= 60 mins	
**Gender, n (%)**			0.203
Male	356 (50.7)	133 (55.6)	
Female	346 (49.3)	106 (44.4)	
**Age in years, n (%)**			<0.001
< 20 years	73 (10.4)	10 (4.2)	
20–40 years	385 (54.8)	119 (49.8)	
40–65 years	186 (26.5)	90 (37.7)	
> 65 years	58 (8.3)	20 (8.4)	
**Marital status, n (%)**			0.001
Single	301 (42.9)	71 (29.7)	
Married	345 (49.1)	148 (61.9)	
Others	56 (8.0)	20 (8.4)	
**Level of education, n (%)**			0.084
None/ Primary	23 (3.3)	8 (3.4)	
Secondary	131 (18.7)	30 (12.6)	
Diploma/ University	548 (78.1)	201 (84.1)	
**Employment status, n (%)**			0.027
Employed/ Self Employed	487 (69.4)	177 (74.1)	
Not Employed	148 (21.1)	34 (14.2)	
Retired	52 (7.4)	26 (10.9)	
Student	15 (2.1)	2 (0.8)	
**Race, n (%)**			0.294
African	635 (90.5)	222 (92.9)	
Others	67 (9.5)	17 (7.1)	
**Day of visit, n (%)**			0.008
Monday	106 (15.1)	59 (24.7)	
Tuesday	72 (10.3)	26 (10.9)	
Wednesday	110 (15.7)	40 (16.7)	
Thursday	104 (14.8)	32 (13.4)	
Friday	111 (15.8)	36 (15.1)	
Saturday	106 (15.1)	30 (12.6)	
Sunday	93 (13.3)	16 (6.7)	
**Time of visit, n (%)**			<0.001
Morning (8am–12 noon)	352 (50.1)	30 (12.6)	
Afternoon (12 noon–4pm)	154 (21.9)	84 (35.1)	
Evening (4pm–10pm)	85 (12.1)	111 (46.4)	
Night (10pm–8am)	111 (15.8)	14 (5.9)	
**Times visit AKUHN, n (%)**			<0.001
None	125 (17.8)	26 (10.9)	
1–2	338 (48.1)	96 (40.2)	
2–5	186 (26.5)	91 (38.1)	
> 5	53 (7.5)	26 (10.9)	
**Time spent with the doctor, n (%)**			0.004
Less than 15 mins	529 (75.4)	157 (65.7)	
More than 15 mins	173 (24.6)	82 (34.3)	
**Overall satisfaction score with wait times, n (%)**			<0.001
Satisfied	684 (97.4)	25 (10.5)	
Neutral	13 (1.9)	84 (35.1)	
Not satisfied	5 (0.7)	130 (54.3)	
**Overall satisfaction score with service times, n (%)**			<0.001
Satisfied	695 (99.0)	143 (59.8)	
Neutral	7 (1.0)	89 (37.2)	
Not satisfied	0 (0.0)	7 (2.9)	
**Likelihood to return for care at AKUHN, n (%)**			<0.001
Always	633 (90.2)	33 (13.8)	
Occasionally	68 (9.7)	129 (54.0)	
Rarely	1 (0.1)	62 (25.9)	
Never	0 (0.0)	15 (6.3)	
**Likelihood to recommend to relatives and friends, n (%)**			<0.001
Always	641 (91.3)	34 (14.2)	
Occasionally	60 (8.5)	130 (54.4)	
Rarely	1 (0.1)	66 (27.6)	
Never	0 (0.0)	9 (3.8)	

Furthermore, we conducted multivariate logistic regression to identify associations of overall satisfaction with prolonged waiting time after adjusting for age, marital status and employment. Participants expressing not satisfied with waiting times had higher odds of having to wait longer as compared to those that expressed neutral satisfaction (OR: 3.70, 95% CI: 1.27–11.02; p = 0.017) whereas those participants expressing satisfied with waiting times had lower odds of having to wait longer as compared to those that expressed neutral satisfaction (OR: 0.005, 95% CI: 0.002–0.010; p < 0.001). Participants expressing satisfied with service times had lower odds of having to wait longer as compared to those that expressed neutral/ not satisfied (OR: 0.014, 95% CI: 0.006–0.032; p < 0.001).

## Discussion

In this study, patients reported waiting times ranging from less than 30 minutes to over 120 minutes. Most patients waited between 31–60 minutes to be seen by a healthcare provider, with nearly a quarter waiting between 61–120 minutes. Only 23.5% of patients were seen within the recommended 30 minutes of arrival. Many patients in our healthcare setting rely on the emergency room as their main entry point into the healthcare system because they don’t have a primary care physician. Our data supports this, as almost a quarter of the visits are follow-up visits. For this study, we have also defined prolonged waiting times as those exceeding 60 minutes [12,15,16].

Long waiting times are a common problem worldwide, affecting both developed and developing countries. Studies conducted in low- and middle-income countries (LMICs) have shown that patients often wait 2–4 hours before seeing a healthcare provider [13,20]. In West Africa, studies conducted in Benin and Nigeria reported average waiting times of over two hour [13,18]. However, studies conducted in South Africa [20] and Ethiopia [8] reported slightly better average waiting times at 69 and 93 minutes, respectively.

In contrast, looking at high-resource countries, a study conducted by Horowitz et al. in the United States found that fewer than one-fifth of ERs could see 90% of their patients within one hour [17]. The waiting times also varied greatly from state to state [17]. Another review by McIntyre et al., which examined turnaround times in Emergency departments as an indicator of Health Services Under Strain, reported that 29% of Canadians reported waiting over 4 hours in emergency departments [21]. Similarly, a study in California ERs showed that patients waited an average of 56 minutes to see a doctor [22]. According to the Australian Institute of Health and Welfare (2017–2018), the median waiting time ranged from 15 to 46 minutes. However, the 90th percentile waiting times varied from 78 to 159 minutes [23]. A study conducted in the United Kingdom showed that patients waited on average for 70 minutes, and longer waiting times in ERs were associated with adverse outcomes [24]. The large variations in waiting times can be attributed to the availability of healthcare resources and the geographic location of the healthcare facility. While waiting times may vary between countries and regions, our study found that waiting times at our institution were similar to those in developed countrie [17,21–23], which could be attributed to better resources available at a private institution compared to a public healthcare facility including better staffing, patient flows and queueing systems.

Most study participants (65.6%) reported that their ER consultation with the healthcare provider lasted about 5–15 minutes. Only 23.7% of patients reported spending 15–25 minutes with the healthcare provider. A study by Ming Tae-Seale et al. in an outpatient ER found that doctors spent at least 13–24 minutes with elderly patients [24]. In our study, among those who were satisfied with the consultation times, the majority waited for 31–60 minutes to see a healthcare provider with a consultation time of 5–15 minutes. However, for those not satisfied with the waiting times, the majority had waited for at least one hour. Similar to other studies, our study also showed that prolonged waiting times negatively affected patient satisfaction and influenced the general perception of the service received by patients [25]. Furthermore, a study by Bleustein and colleagues found that prolonged waiting times impacted how patients perceived information and instructions and the overall treatment provided by the care team. The authors noted that the patient experience was heavily influenced by time spent waiting to receive care, and every aspect of the care experience, including confidence in the care provider and perceived quality of care, were negatively affected by prolonged waiting times in hospitals.

According to our study, patients visiting the emergency room on Mondays and Wednesdays had to wait longer compared to the other days of the week. This could be attributed to patients visiting the ER more often after the weekend, leading to more visits. With a fixed staffing model, higher patient volumes are expected to lead to prolonged waiting times. Our findings are similar to a study conducted in the Jimna zone of Southwest Ethiopia [8], which found that patients who visited on Mondays were more likely to wait longer than the rest of the week. Moreover, the study also noted that patients who visited in the morning were more likely to wait longer than those who visited in the afternoon.

We also found that the chances of experiencing prolonged wait times in the ER were highest between 4 pm and 10 pm, compared to those who visited during morning hours (8.00 am-noon). Patients who visited the ER between 4 pm and 10 pm were more likely to wait longer to see a healthcare provider than those who visited in the morning. Our findings suggest that patients visit the ER after working hours, which corresponds to when the ER is minimally staffed.

We also found that prolonged waiting times were associated with specific demographic characteristics such as age, marital status, and employment status. Almost half of the participants who experienced a lengthy waiting time were above 40 years of age (46.1%). This is consistent with a study conducted in Riyadh, Saudi Arabia, where waiting times for individuals aged 65 years and above were significantly longer than for younger age groups [26]. This could be attributed to the higher scrutiny and triage given to the older population due to their medical complexity and the increased likelihood of having multiple medical conditions.

The patient’s employment status also seemed to be related to waiting times, where patients with a higher socioeconomic status tended to experience longer waiting times and overall dissatisfaction with the service provided at the ER. Of interest, a study conducted in Nigeria found that patients with a higher socioeconomic status were more likely to demand shorter waiting times and report negative patient experiences [27]. Additionally, our findings are similar to those of Maharlouei et al. and Arpey et al., who found that patients with better socioeconomic status were more dissatisfied with the services provided by the healthcare system [28,29]. These factors can lead to unconscious bias by physicians, which can affect their clinical judgments and eventually result in disparities in the quality of care, as shown in multiple studies [30–32]. This could be explained by the fact that employed persons are more likely to visit the ER after working hours when the ER is not adequately staffed. It is also possible that patients from a higher socioeconomic status had unrealistic expectations of receiving better and quicker service in the emergency room.

In our study, most participants said they would return to the hospital for care and recommend the ER services to their family and friends. However, 8.3% and 8.1% of the participants were unwilling to return for care or recommend the hospital to others due to long waiting times. This is consistent with other studies which showed that customers and patients who experience long waiting times tend to be dissatisfied and are less likely to return for care. This can lead to a long-term loss of profits for the organisation, as dissatisfied customers are more likely to convey their dissatisfaction to others [33–36]. These studies recommended using updated technology, adequate staffing, and patient-centred strategies to help reduce waiting times.

Our study highlights certain key factors that can be implemented in LMIC countries such as Kenya. Firstly, to focus on building primary care services that can alleviate the pressures and waiting times in the ER and allow patients to obtain longitudinal care in an appropriate clinical setting. Secondly, to explore the concept of Urgent Care Centers (analogous to those found in higher income countries) within heavily populated areas that can provide care to non-emergent patients in order to minimize waiting times and improve satisfaction. Thirdly, in resource limited settings, it is important to staff the ER based on their patient volumes and patient characteristics. In our case this would entail adequately staffing the ER on Mondays (after the weekend) and between 4pm to 10 pm (after working hours). By targeting staffing availability to patient needs one could hope to improve the patient experience, decrease wait times and improve overall satisfaction. Lastly studies assessing wait times and patient satisfaction should be conducted routinely in order to assess whether interventions such as those suggested above are impactful and sustainable.

Like other studies, our study also has several limitations. Firstly, we should have considered overall turnaround times as patients were not asked about their time in other ancillary services such as the laboratory, pharmacy and radiology, which could have affected their overall waiting time and services. Secondly, we only focused on patients seeking care in the ER and did not include patients treated at other outpatient speciality clinics. We used a convenience sample for recruitment with its known limitations. We also did not question patients on why they were either satisfied or dissatisfied with the service and wait times in the ER. Thirdly, waiting times were an approximation from the study participants and could not be fully verified via a queue system. Furthermore, even though the surveys were anonymous, patients could have felt that answering the questions might have affected their overall care in the ER. Fourthly, we included people accompanying patients in the survey however we did not state the number of patients versus the accompanying adults and their experience and satisfaction may have differed. In addition, we did not assess the staffing levels during data collection or the relationship between the number of healthcare providers and waiting times. Lasty, while machine learning methods for analyzing wait times, time series and prediction of satisfaction have shown promise in various studies [37–39], this study used a manual method to collect data and a traditional method of data analyses, and we acknowledge this as a limitation. Machine learning techniques have been shown to improve predictive accuracy and uncover patterns in wait time data that may not be easily captured through traditional statistical methods. Machine models usually require large sets of data to provide a more accurate prediction model, which we lacked during the study period. Future research could explore integrating these advanced techniques to refine predictive models and provide deeper insights into ER wait time fluctuations, ultimately supporting more efficient resource allocation and patient flow management.

## Conclusion

Patient waiting time and ER experiences can influence the patient’s perception of care. In this study, prolonged waiting times were linked to patient dissatisfaction and had a negative impact on the quality of care. Healthcare institutions in LMICs must devote more time and resources to address prolonged waiting times in key clinical areas such as the ER. Identifying bottlenecks and gridlocks within healthcare facilities is essential to develop an effective plan to reduce waiting times and improve care delivery. This will ultimately translate into improved patient satisfaction and outcomes. Some mitigating strategies include queueing systems, comprehensive health information systems, staffing according to demands and needs, and improving patient flows.

## Supporting information

S1 Data PLOS ONE DATA - WAITING TIME.xlsx
(XLSX)

## References

[1] Xie Z, Or C. Associations between Waiting Times, Service Times, and Patient Satisfaction in an Endocrinology Outpatient Department: A Time Study and Questionnaire Survey. Inquiry. 2017;54:0046958017739527. doi: 10.1177/0046958017739527 29161947 PMC5798665

[2] Ferreira DC, Vieira I, Pedro MI, Caldas P, Varela M. Patient Satisfaction with Healthcare Services and the Techniques Used for its Assessment: A Systematic Literature Review and a Bibliometric Analysis. In: Healthcare. MDPI; 2023. p. 639. 10.3390/healthcare11050639 PMC10001171 36900644

[3] Xesfingi S, Vozikis A. Patient satisfaction with the healthcare system: Assessing the impact of socio-economic and healthcare provision factors. BMC Health Serv Res. 2016;16(1):1–7. 26979458 10.1186/s12913-016-1327-4 PMC4793546

[4] Ahmad I, Nawaz A, Khan S, Khan H, Rashid MA, Khan MH. Predictors of patient satisfaction. Gomal Journal of Medical Sciences. 2011;9(2).

[5] Prakash B. Patient satisfaction. J Cutan Aesthet Surg. 2010;3(3):151–5. doi: 10.4103/0974-2077.74491 21430827 PMC3047732

[6] Huang J-A, Lai C-S, Tsai W-C, Weng R-H, Hu W-H, Yang D-Y. Determining factors of patient satisfaction for frequent users of emergency services in a medical center. J Chin Med Assoc. 2004;67(8):403–10. 15553800

[7] Bleustein C, Rothschild DB, Valen A, Valatis E, Schweitzer L, Jones R. Wait times, patient satisfaction scores, and the perception of care. Am J Manag Care. 2014;20(5):393–400. 25181568

[8] Biya M, Gezahagn M, Birhanu B, Yitbarek K, Getachew N, Beyene W. Waiting time and its associated factors in patients presenting to outpatient departments at Public Hospitals of Jimma Zone, Southwest Ethiopia. BMC Health Serv Res. 2022;22(1):107. doi: 10.1186/s12913-022-07502-8 35078474 PMC8790858

[9] Zhu X, Hu Y, Wang L, Li D, Wu X, Xia S, et al. An Observational Study of Physicians’ Workflow Interruptions in Outpatient Departments in China. Front Public Health. 2022;10:884764. doi: 10.3389/fpubh.2022.884764 35757627 PMC9215343

[10] Manary MP, Boulding W, Staelin R, Glickman SW. The patient experience and health outcomes. N Engl J Med. 2013. 10.1056/NEJMp1211775 23268647

[11] Ashton F, Hamid K, Sulieman S, Eardley W, Baker P. Factors influencing patient experience and satisfaction following surgical management of ankle fractures. Injury. 2017;48(4):960–5. doi: 10.1016/j.injury.2017.02.017 28249677

[12] O’Malley MS, Fletcher SW, Fletcher RH, Earp JA. Measuring patient waiting time in a practice setting: a comparison of methods. J Ambul Care Manage. 1983;6(3):20–7. doi: 10.1097/00004479-198308000-00006 10260999

[13] Enabulele O, Ajokpaniovo J, Enabulele JE. Patient waiting and consultation time in the general practice clinic of the University of Benin teaching hospital, Edo State, Nigeria. J Family Med Community Health. 2018;5(2):1146.

[14] Wamai RG. The Kenya Health System—Analysis of the situation and enduring challenges. Jmaj. 2009;52(2):134–40.

[15] Beveridge R. Canadian emergency department triage and acuity scale: implementation guidelines. CJEM. 1999;1:S2–28.

[16] Murray M, Bullard M, Grafstein E. Revisions to the Canadian emergency department triage and acuity scale implementation guidelines. Can J Emerg Med. 2004;6(6):421–7. doi: 10.1017/s1481803500009428 17378961

[17] Horwitz LI, Green J, Bradley EH. US emergency department performance on wait time and length of visit. Ann Emerg Med. 2010;55(2):133–41. doi: 10.1016/j.annemergmed.2009.07.023 19796844 PMC2830619

[18] Dansky KH, Miles J. Patient satisfaction with ambulatory healthcare services: waiting time and filling time. Journal of Healthcare Management. 1997;42(2):165–77. 10167452

[19] Harris PA, Taylor R, Minor BL, Elliott V, Fernandez M, O’Neal L, et al. The REDCap consortium: building an international community of software platform partners. J Biomed Inform. 2019;95:103208. doi: 10.1016/j.jbi.2019.103208 31078660 PMC7254481

[20] Rabie T, Swart A-T, Muller CE. The role of triage to reduce waiting times in primary health care facilities in the North West province of South Africa. Health SA Gesondheid. 2018;23(1):1–6. doi: 10.4102/hsag.v23i0.1097 31934386 PMC6917407

[21] McIntyre D, Chow CK. Waiting time as an indicator for health services under strain: a narrative review. Inquiry. 2020;57:0046958020910305. doi: 10.1177/0046958020910305 32349581 PMC7235968

[22] Lambe S, Washington DL, Fink A, Laouri M, Liu H, Fosse JS, et al. Waiting times in California’s emergency departments. Ann Emerg Med. 2003;41(1):35–44. doi: 10.1067/mem.2003.2 12514681

[23] Duggan M, Harris B, Chislett WK, Calder R. Nowhere else to go: Why Australia’s health system results in people with mental illness getting ‘stuck’ in emergency departments. 2020.

[24] Tai-Seale M, McGuire TG, Zhang W. Time allocation in primary care office visits. Health Serv Res. 2007;42(5):1871–94. doi: 10.1111/j.1475-6773.2006.00689.x 17850524 PMC2254573

[25] Molalign Takele G, Abreha Weldesenbet N, Girmay N, Degefe H, Kinfe R. Assessment patient satisfaction towards emergency medical care and its determinants at Ayder comprehensive specialized hospital, Mekelle, Northern Ethiopia. PLoS One. 2021;16(1):e0243764. doi: 10.1371/journal.pone.0243764 33411806 PMC7790252

[26] Elkum N, Fahim M, Shoukri M, Al-Madouj A. Which patients wait longer to be seen and when? A waiting time study in the emergency department. East Mediterr Health J. 2009;15(2):416–424. doi: 10.26719/2009.15.2.416 19554989

[27] Aloh HE, Onwujekwe OE, Aloh OG, Okoronkwo IL, Nweke CJ. Impact of socioeconomic status on patient experience on quality of care for ambulatory healthcare services in tertiary hospitals in Southeast Nigeria. BMC Health Serv Res. 2020;20(1):1–9. doi: 10.1186/s12913-020-05332-0 32456633 PMC7251830

[28] Maharlouei N, Akbari M, Akbari M, Lankarani KB. Socioeconomic status and satisfaction with public healthcare system in Iran. Int J Community Based Nurs Midwifery. 2017;5(1):22. 28097175 PMC5219562

[29] Arpey NC, Gaglioti AH, Rosenbaum ME. How socioeconomic status affects patient perceptions of health care: a qualitative study. J Prim Care Community Health. 2017;8(3):169–75. doi: 10.1177/2150131917697439 28606031 PMC5932696

[30] Bernheim SM, Ross JS, Krumholz HM, Bradley EH. Influence of patients’ socioeconomic status on clinical management decisions: a qualitative study. Ann Fam Med. 2008;6(1):53–9. doi: 10.1370/afm.749 18195315 PMC2203396

[31] Ross JS, Bradley EH, Busch SH. Use of health care services by lower-income and higher-income uninsured adults. JAMA. 2006;295(17):2027–36. doi: 10.1001/jama.295.17.2027 16670411

[32] Bernheim SM, Spertus JA, Reid KJ, Bradley EH, Desai RA, Peterson ED, et al. Socioeconomic disparities in outcomes after acute myocardial infarction. Am Heart J. 2007;153(2):313–9. doi: 10.1016/j.ahj.2006.10.037 17239695

[33] Al-Sakkak MA, Al-Nowaiser NA, Al-Khashan HI, Al-Abdrabulnabi AA, Jaber RM. Patient satisfaction with primary health care services in Riyadh. Saudi Med J. 2008;29(3):432–6. 18327374

[34] Mohamed W, Swat K, Wahab M, Alsulaimani A, Portugal A. Patient satisfaction: a comparison between governmental and private out-patient clinics in Taif, Saudi Arabia. Madridge J Case Rep Stud. 2017;1(1):1–6. doi: 10.18689/mjcrs-1000101

[35] Llego J, Al Shirah M. Patient satisfaction in tertiary private hospitals in Najran, Kingdom of Saudi Arabia. Int J Res Foundation Hosp Healthc Adm. 2017;5(1):42–6.

[36] Tan WS, Chua SL, Yong KW, Wu TS. Impact of pharmacy automation on patient waiting time: an application of computer simulation. Ann Acad Med Singap. 2009;38(6):501. doi: 10.47102/annals-acadmedsg.v38n6p501 19565100

[37] Curtis C, Liu C, Bollerman TJ, Pianykh OS. Machine learning for predicting patient wait times and appointment delays. J Am Coll Radiol. 2018;15(9):1310–6. doi: 10.1016/j.jacr.2017.08.021 29079248

[38] Joseph J, Senith S, Kirubaraj AA, Ramson JSR. Machine Learning for Prediction of Wait Times in Outpatient Clinic. Procedia Comput Sci. 2022;215:230–9.

[39] Pak A, Gannon B, Staib A. Predicting waiting time to treatment for emergency department patients. Int J Med Inform. 2021;145:104303. doi: 10.1016/j.ijmedinf.2020.104303 33126060

